# Physics of mechanotransduction by Piezo ion channels

**DOI:** 10.1085/jgp.202113044

**Published:** 2022-05-20

**Authors:** Michael Young, Amanda H. Lewis, Jörg Grandl

**Affiliations:** 1 Department of Neurobiology, Duke University Medical Center, Durham, NC

## Abstract

Piezo ion channels are sensors of mechanical forces and mediate a wide range of physiological mechanotransduction processes. More than a decade of intense research has elucidated much of the structural and mechanistic principles underlying Piezo gating and its roles in physiology, although wide gaps of knowledge continue to exist. Here, we review the forces and energies involved in mechanical activation of Piezo ion channels and their functional modulation by other chemical and physical stimuli including lipids, voltage, and temperature. We compare the three predominant mechanisms likely to explain Piezo activation—the force-from-lipids mechanism, the tether model, and the membrane footprint theory. Additional sections shine light on how Piezo ion channels may affect each other through spatial clustering and functional cooperativity, and how substantial functional heterogeneity of Piezo ion channels arises as a byproduct of the precise physical environment each channel experiences. Finally, our review concludes by pointing out major research questions and technological limitations that future research can address.

## Teaser

Young et al. review and synthesize the mechanistic principles of forces and energies involved in Piezo ion channel mechanotransduction.

## Energetics of mechanosensitive ion channel gating: A framework

Ion channels, which facilitate the movement of ions down their electrochemical gradient and across membranes, require energy to transition between conformational states. Piezo ion channels are no exception: they gate (open and close) in response to mechanical forces, allowing the nonselective flux of cations into the cell (Coste et al., 2010; Coste et al., 2012). The difference in free energy (Δ*G*_0_) between the open (conducting) and closed (nonconducting) states of the Piezo protein, not including the membrane, is

$$\Delta G_{0}=G_{open}-G_{closed}\text{.}$$

The probability of finding Piezos in the open state (*P*_*o*_) depends on Δ*G*′, which is the difference between Δ*G*_0_ and the sum of all combined external energies (*G*_*j*_ = *G*_*coulombic*_, *G*_*ligand*_, *G*_*thermal*_, etc.):

$$∆G^{\prime }=∆G_{0}-\sum G_{j}\text{,}$$

$$P_{o}=\frac{1}{1+\exp\left(\frac{{\Delta G}^{\prime }}{k_{B}∙T}\right)}\text{.}$$

For Piezos, the open state is higher energy compared with the closed state, and Δ*G*_0_ is positive. Consequently, the basal open probability of Piezos is low. When external energy is added, Δ*G*′ is reduced (or even made negative), and *P*_*o*_ is increased. There are many potential sources of external energy, including mechanical energy (*G*_*mech*_), which is the focus of our review.

Importantly, many ion channels efficiently couple to multiple sources of external energy, making them polymodal. For example, in TRPV1, opening can be driven by voltage, temperature, and the chemical ligand capsaicin (Julius, 2013; Voets et al., 2004; Latorre et al., 2007). Specifically, hot temperatures and capsaicin can act either individually or in concert to dramatically shift the voltage dependence of TRPV1 to a physiological range—i.e., the modalities are allosterically coupled to reduce Δ*G*_0_. Similarly, large conductance BK potassium channels are allosterically gated by both Ca^2+^ ions and depolarizing potentials (Horrigan and Aldrich, 2002).

Consistent with this concept, all membrane proteins are, in principle, sensitive to mechanical energy. For example, stretching of the membrane bilayer can weakly shift the voltage dependence of voltage-gated ion channels (Beyder et al., 2010; Morris, 2011; Schmidt et al., 2012). However, for most channels, the contribution of mechanical energy to gating will be negligible, i.e., *G*_*mech*_ ≪ Δ*G*_0_.

**Figure 1.**
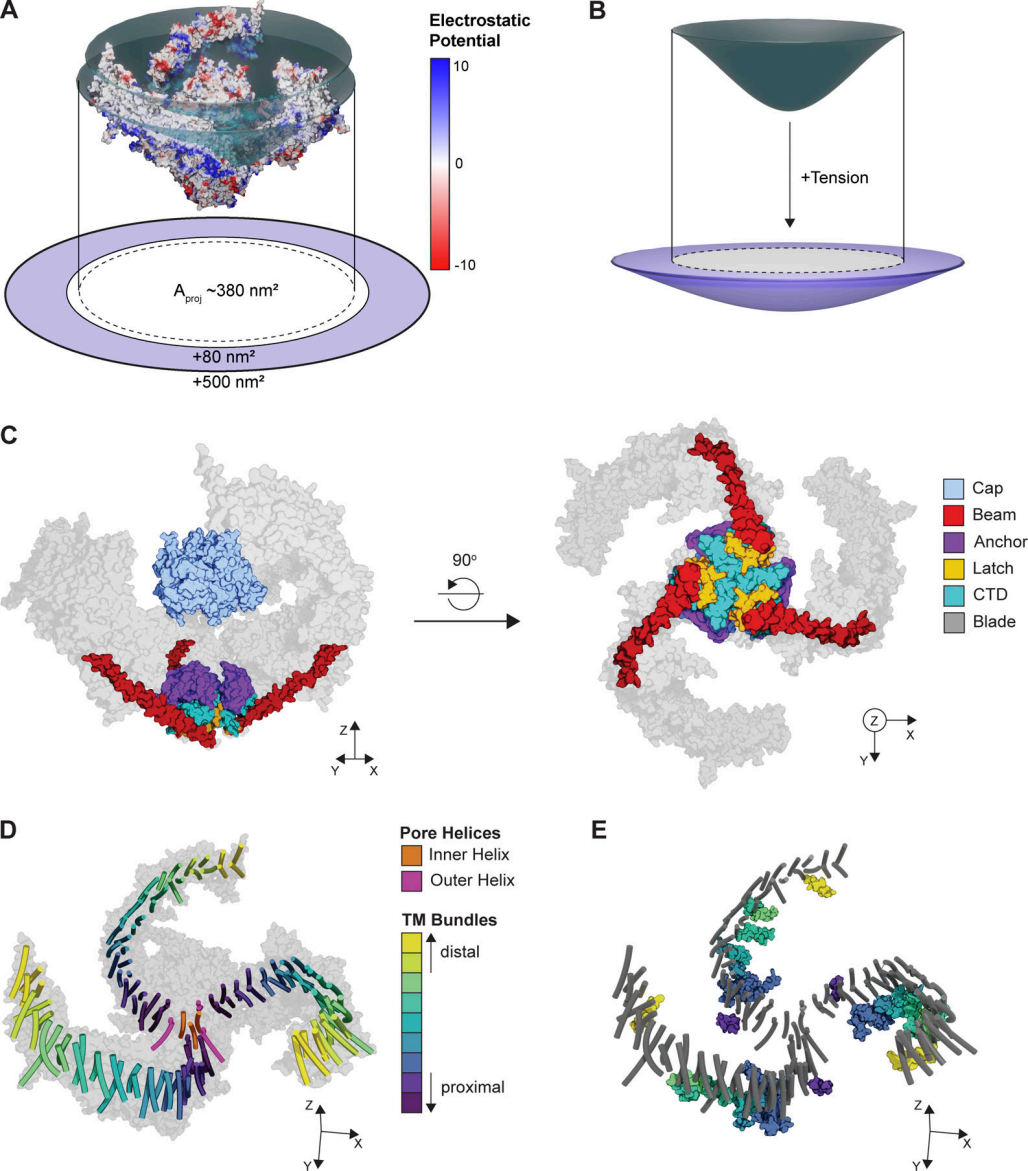
**Structure of Piezo ion channels. (A)** Surface model of mouse Piezo2 (PDB accession no. 6KG7) colored according to electrostatic potential and embedded in a disc model of the putative membrane plane (dark teal). Red indicates a negative potential, and blue indicates a positive potential. The discs highlight the curvature of the uncharged region of the TMs. The projected area (A_proj_) of the channel is shown as a dotted line. The putative range of area expansion upon flattening (80–500 nm) of the Piezo protein is shown in light purple. **(B)** Top: Side view of the membrane deformation in A (dark teal). Bottom: Schematized area expansion in the presence of tension with projected area of the relaxed membrane configuration overlaid (white + dotted line). **(C)** Left: Side view of a space-filling model of the Piezo2 cap, anchor, latch, beam, and C-terminal domain (CTD). Right: Bottom view of the same structures. A space-filling model of the blades is shown in light gray. **(D)** Transmembrane helix (TM) organization of the Piezo2 structure. TMs are colored according to bundle, which each containing four TMs. A space-filling model of the full protein is shown in light gray. **(E)** Same structure as in D, additionally showing interbundle amphipathic helices, colored according to the preceding bundle as in D. TMs are shown in dark gray.

In this review, we focus on the energetics of Piezo ion channels (Box 1 and Fig. 1), which are, in essence, “professional mechanosensors”: they are activated directly by mechanical stimuli with exquisite sensitivity but are nearly inert to voltage, temperature, pH, and ligands. Upon opening, Piezos become permeable to cations, including calcium, and rapidly inactivate in response to prolonged stimuli. Together, these properties make them ideal proteins for transducing mechanical energy into electrochemical signals and mediating diverse mechanotransduction processes throughout the body (Coste et al., 2010; Coste et al., 2012; Kefauver et al., 2020). The specialization of Piezos as mechanosensors means that mechanical energy alone is sufficient to substantially reduce Δ*G*_0_, and it can therefore drive gating transitions through the entire range of open probabilities. In contrast, charge movement, ligand binding, and temperature contribute relatively little energy (*G*_*coulombic*_, *G*_*ligand*_, *G*_*thermal*_ ≪ Δ*G*_0_) and therefore only weakly modulate Piezo open probability.

**Box 1: General architecture of Piezos**

In vertebrates, there are two isoforms, Piezo1 and Piezo2, that share significant homology to each other, but little homology to other membrane proteins. Cryo-EM structures reveal that both proteins are trimeric, with each subunit comprising 38 TMs (Wang et al., 2019; Guo and Mackinnon, 2017; Saotome et al., 2018). The most complete structure to date is of mouse Piezo2 (Wang et al., 2019) and reveals that the first 36 TMs form the “blades” of the protein and are arranged in nine bundles of four TMs each that spiral out from a central pore in a highly curved, triskelion-like shape that forms a dome in the cell membrane (Fig. 1, A and D). Except for the most distal bundle, each is preceded by an amphipathic helix that lies parallel to the membrane (Fig. 1 E). The final two TMs, termed inner helix (IH) and outer helix (OH), line the central pore, which is permeant to cations including calcium. The pore is coupled in a domain-swapped fashion to an extracellular cap that, at least in existing structures, is embedded in the center of the blades (Fig. 1 C). The pore domain is also connected to the extended blades by a long, continuous α helix, termed the beam, that runs intracellularly from the pore to the third-most proximal bundle in each blade. The beam terminates in a latch domain that connects to the pore via a C-terminal intracellular domain and additionally interfaces with a triangle-shaped anchor domain that is wedged between the pore and the first helical bundle, as well as plug domains that may regulate ion permeation through intracellular portals (Taberner et al., 2019; Geng et al., 2020). The unique, curved shape of Piezos can induce significant curvature in lipid bilayers (Lin et al., 2019), which can be appreciated by visualizing the electrostatic surface potential, which is largely neutral in the transmembrane regions of the protein (Fig. 1 A).

In principle, mechanical energy can arise from multiple sources which, importantly, are not mutually exclusive (Haswell et al., 2011). While the two best described mechanisms for mechanical activation are “force-from-lipids” (see The force-from-lipid model) and “force-from-tether” (see The force-from-tether model), the unique size and structure of Piezo channels has led to the additional hypothesis of a “footprint mechanism” (see The membrane footprint model), which also predicts potential spatial and functional cooperativity (see Piezo channel cooperativity). Under physiological conditions, thermal energy, voltage, and ligands do not activate Piezos, but are nevertheless important functional modulators (see Modulation of Piezo mechanosensitivity…). The local environment also represents an important source of functional modulation and heterogeneity (see Heterogeneity of mechanical gating). In the following sections, we discuss what is known, and what remains to be discovered (see Looking forward: Future directions…) regarding the contribution of different energy sources to gating of Piezo ion channels.

## The force-from-lipid model

**Figure 2.**
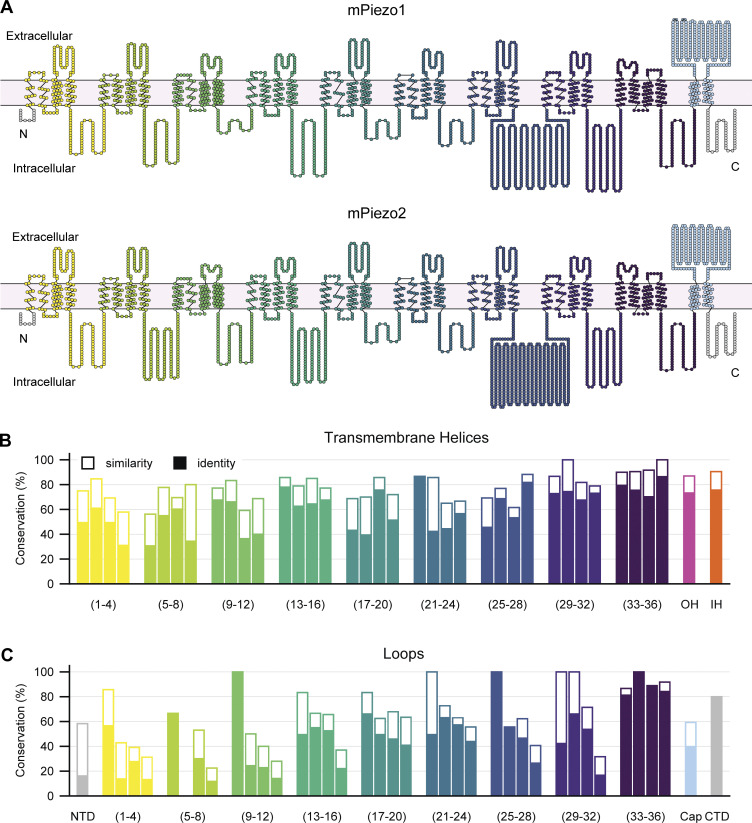
**Comparison between mouse Piezo1 and mouse Piezo2. (A)** Top: Snake plot of mouse Piezo1 (Uniprot accession no. E2JF22) illustrating the TMs and loops. Each circle represents one amino acid. Bottom: Identical snake plot of mouse Piezo2 (Uniprot accession no. Q8CD54). Helical bundles are colored as in Fig. 1 A. Interbundle loops are colored according to the preceding bundle. The N- and C-terminal loops are shown in gray, the outer helix is shown in pink, the inner helix is shown in orange, and the cap domain is shown in light blue. **(B)** Conservation of individual TMs between mouse Piezo1 and mouse Piezo2. Bars are organized into bundles and colored as in A. Amino acid identity is shown in filled bars, and similarity is shown in outlined bars for each helix. **(C)** Same as in B, but for all loops.

In the force-from-lipid model, mechanical energy is provided in the form of membrane tension (γ)—a 2D force propagated through the lipid bilayer to the channel with no requirement for other cellular components. Importantly, force-from-lipids alone is sufficient to activate Piezo1 (see Box 2 and Fig. 2 for comparison of Piezo1 versus Piezo2). Specifically, work from several laboratories showed that Piezo1 is directly activated by lateral membrane tension, an intact cytoskeleton is not required for channel activity, and most importantly, Piezo1 can be reconstituted and activated by tension in a cell-free lipid bilayer system (Lewis and Grandl, 2015; Cox et al., 2016; Syeda et al., 2016).

**Box 2: Piezo1 versus Piezo2**

Overall, the structures of Piezo1 and Piezo2 are quite similar: at the amino acid level, they share ∼40% sequence homology and have an identical overall architecture, with particularly strong conservation in transmembrane helices compared to loops and overall increasing conservation toward the C-terminus (Saotome et al., 2018; Wang et al., 2019; Guo and Mackinnon, 2017; Fig. 2, A–C). In principle, the similar structures suggest the two proteins should have similar activation mechanisms and gating energies, particularly if the predominant force is applied through the membrane alone. However, several key differences exist between the two proteins that suggest the energetic contributions to their gating may differ.

Piezo1 and Piezo2 proteins have similar thresholds in the poke indentation assay as well as similar macroscopic current amplitudes (Coste et al., 2010; Wu et al., 2017; Taberner et al., 2019). However, whereas Piezo1 is robustly activated by the negative pressure-clamp assay, Piezo2 is not: only a fraction of transfected cells responds at all, and the few responders typically have small currents (Moroni et al., 2018; Coste et al., 2015; Ikeda and Gu, 2014; Shin et al., 2019; Verkest et al., 2022). The similar shape of the two proteins suggests that both, in principle, should be sensitive to gating energies of the membrane footprint. Yet if they are, why would increases in lateral tension achieved by the pressure clamp system not efficiently activate Piezo2? Perhaps Piezo2 is less sensitive to membrane tension and/or direct activation through force-from-lipids, and instead relies more on gating through a tether mechanism.

Consistent with this idea, Piezo2 is less sensitive to margaric acid (which stiffens the cell membrane and increases the gating threshold for Piezo1) and more sensitive to latrunculin A (which prevents actin polymerization) than is Piezo1 (Romero et al., 2019; Romero et al., 2020). The differential sensitivity is linked to the beam domain in each protein, suggesting this as a possible site of differential cytoskeletal tethering between the two proteins. Additionally, Piezo2 peak current amplitudes are insensitive to temperature, whereas Piezo1 peak currents are inhibited (Zheng et al., 2019b). Together, these lines of evidence suggest that force-from-lipid may not be the primary activation mechanism for Piezo2, as cooling is expected to stiffen the membrane and potentially reduce transmission of force (Pan et al., 2008). Piezo2 is also insensitive to Yoda1: this could result from heterogeneity in the proposed binding site, but also from differences in how the blades transmit force to the channel pore (Lacroix et al., 2018; Botello-Smith et al., 2019). On the other hand, the spider toxin GsMTx4, which nonspecifically inhibits mechanically activated ion channels by distorting local tension and reducing the efficiency of transfer to the channel, inhibits both Piezo1 and Piezo2 (Gnanasambandam et al., 2017; Alcaino et al., 2017). Future experiments to test whether Piezo2 can be activated in a cell-free environment are critical to clarify some of these discrepancies.

As described earlier, the difference in energy between the closed and open conformations of the Piezo protein is Δ*G*_0_. An increase in membrane tension (γ) induces a conformational change that includes an area expansion (Δ*A* = *A*_*open*_ − *A*_*closed*_) that can overcome Δ*G*_0_. Therefore, the total Gibbs free energy (Δ*G*′) of this system is

$$∆G^{\prime }=∆G_{0}-\gamma ∙\Delta A\text{.}$$

Differentiating this equation with respect to tension reveals that the tension sensitivity of a channel is directly proportional to its change in cross-sectional area:

$$\frac{\partial ∆G^{\prime }}{\partial \gamma }=-\Delta A\text{.}$$

**Figure 3.**
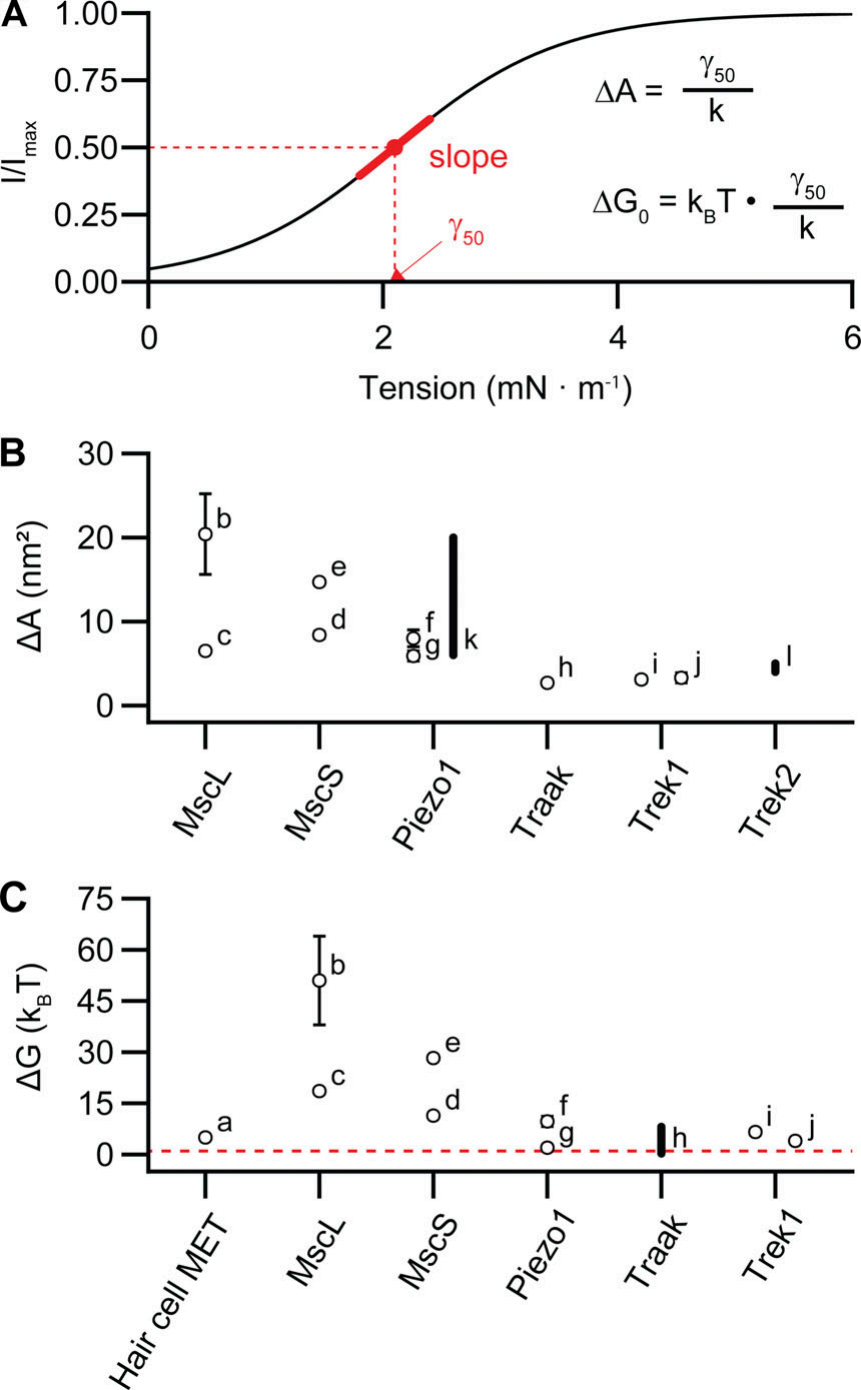
**Estimated area expansions and gating energies of mechanotransduction channels. (A)** Schematized depiction of a sigmoidal current-tension relationship, generated with the equation $\frac{I}{I_{\max}}=\frac{1}{1+e^{\frac{\gamma_{50}-\gamma }{k}}}\text{.}$ The tension of half-maximal activation (γ_50_) and slope factor (*k*) of the sigmoid can be used to estimate the biophysical parameters of channel area expansion (Δ*A*) and gating energy (Δ*G*). **(B and C)** Estimates of channel area expansion upon opening (B) and estimates of channel gating energies for mechanosensitive ion channels (C). The red dotted line represents thermal energy (*k*_*B*_*T*) at 22°C. The preparations from which these values were estimated are as follows: (a) meta-analysis (Ricci et al., 2006), (b) spheroplasts (Chiang et al., 2004), (c) liposomes (Sukharev et al., 1999), (d) liposomes (Sukharev, 2002), (e) spheroplasts (Belyy et al., 2010), (f) cell-attached (Cox et al., 2016), (g) cell-attached with positive pressure prepulse (Lewis and Grandl, 2015), (h) structure (Brohawn et al., 2014a), (i) cell-attached and (j) bleb-attached (Maksaev et al., 2011), (k) cell-attached (Bae et al., 2013), and (l) simulation (Aryal et al., 2017). Error bars are included when uncertainty was reported. Gating energies and area expansions reported as bands are represented as thick lines.

**Figure 4.**
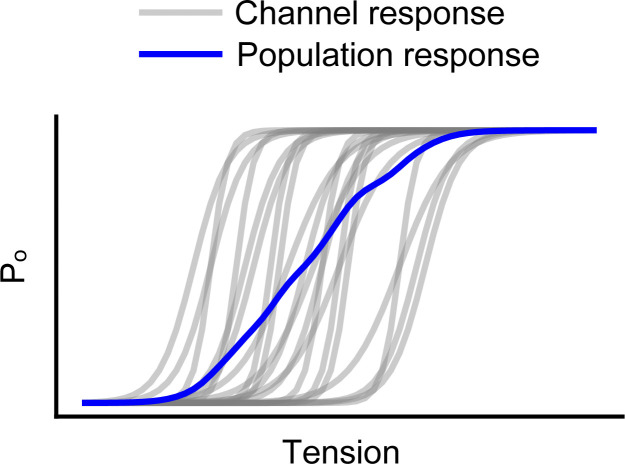
**Broadening of the population tuning curve for mechanotransduction channels.** Simulated equilibrium open probability (*P*_*o*_) as a function of tension for hypothetical individual channels (gray) as well as their average population response (blue). Measurements of channel population activity will necessarily underestimate sensitivity (slope), resulting in a broadening of the tension-response profile.

In other words, a steep slope of the *P*_*o*_–tension relationship reflects a large change in cross-sectional area. This relationship has been well established for the prototypical bacterial mechanosensitive MscL, in which a comparison of crystal structures in open and closed states reveals that it undergoes significant area expansion upon opening (Δ*A* = ∼20 nm^2^; Sukharev et al., 2001; Fig. 3). Area expansion can also be estimated from patch-clamp recordings, in which pressure steps are applied to the patch to induce tension, and peak current amplitude is used as a readout of open probability (Chiang et al., 2004). Indeed, electrophysiology experiments with MscL and Piezo1 expressed in the same patch revealed current–pressure curves with different half-maximal values for activation but similar slopes. Under the assumption that tension scales linearly with pressure, the similar slope values imply that the area expansion of the two proteins is of similar magnitudes (Δ*A* for Piezo1 = 6–20 nm^2^; Cox et al., 2016; Bae et al., 2013; Fig. 3). Consistent with this result, combined patch-clamp and membrane imaging experiments, which allow for a direct conversion of pressure to tension via Laplace’s law, yielded current–tension relationships with a maximal slope factor value (*k*) of 0.7 ± 0.1 mN/m for Piezo1, which provides an estimate for the area expansion for Piezo1 of

$$∆A=\frac{k_{B}T}{k}=\frac{1.38\cdot 10^{-23}\frac{N∙m}{K}\cdot 300K}{0.7\pm 0.1\frac{mN}{m}}=5.9\pm 0.6\;nm^{2}$$

(Lewis and Grandl, 2015). Notably, these values are potentially an underestimate of the true area expansion, as local variance in membrane environment may lead to artificially shallow pressure–response curves for macroscopic Piezo1 currents (Fig. 4; discussed further in Heterogeneity of mechanical gating). Indeed, structural data indicate that Piezos may have a particularly high potential for area expansion: in the absence of force, the Piezo1 dome has a very large surface area of 460 nm^2^, which, owing to its extreme curvature, projects on a smaller in-plane area of ∼380 nm^2^ (see Box 1 and Fig. 1 for a structural overview). Under force, atomic force microscopy experiments revealed that dome flattening causes an increase in the cross-sectional area to ~900 nm^2^. Together, these data led to an estimated Δ*A* of ∼80–500 nm^2^; similar values are predicted for Piezo2 based on the full structure (Wang et al., 2019; Lin et al., 2019; discussed further in The membrane footprint model).

Current–tension relationships can also be used to calculate Δ*G*_0_, which is proportional to the relationship between the half-maximal pressure for activation (γ_50_) and the slope factor (*k*):

$$∆G_{0}=k_{B}T∙\frac{\gamma_{50}}{k}\text{.}$$

For Piezo1, Δ*G*_0_ has been estimated at 2–10 *k*_*B*_*T* (Lewis and Grandl, 2015; Cox et al., 2016; Fig. 3). This contrasts with the much higher gating energies required to overcome Δ*G*_0_ and open MscL (20–50 *k*_*B*_*T*) but is comparable to those required for the mechanosensitive two-pore potassium channels (K2Ps) Trek-1 and TRAAK (∼0.3–7 *k*_*B*_*T*; Chiang et al., 2004; Sukharev et al., 1999; Maksaev et al., 2011; Brohawn et al., 2014a).

These calculations also give insight into the mechanical specialization of each channel type: while MscL has a high sensitivity to tension (steep slope and large Δ*A*), its large Δ*G*_0_ means that a high tension is required to open the channel—consistent with its role as an emergency osmotic pressure release valve that is permeable to large ions (Chiang et al., 2004). In contrast, K2Ps undergo a smaller area expansion (Δ*A* = 2.7 nm^2^ for TRAAK; Brohawn et al., 2014a) and retain a highly selective pore. The gating energy for K2Ps is also much smaller, which may contribute to their polymodality: in addition to mechanical stretch, voltage, temperature, pH, and ligand binding are also capable of overcoming this energy barrier (Honore, 2007). Piezos, remarkably, have both a low threshold for activation (small γ_50_) and a high tension sensitivity (steep slope and large Δ*A*)—which together makes them exquisitely sensitive to small perturbations in tension.

Interactions between the forces within a membrane and the protein residing in it are complex, leading to multiple hypotheses as to how force-from-lipids might couple to area expansion and Piezo channel opening. First, the tension profile in the transmembrane bilayer is complex and asymmetric, including both attractive and repulsive forces, and this asymmetry is further increased under tension (Martinac et al., 2018). For example, K2P channels have been demonstrated to primarily sense tension in the outer leaflet of the bilayer (Clausen et al., 2017). Likewise, reconstituted Piezos are constitutively active in asymmetric droplet bilayers (Coste et al., 2012; Syeda et al., 2016). Further, Piezos are inhibited by translocation of phosphatidylserine from the inner to the outer leaflet; additional experiments with systematically altered membrane lipid composition may help elucidate the underlying mechanism (Tsuchiya et al., 2018). Second, membrane tension causes the bilayer to thin, which may result in tilting or distortion of transmembrane domains (TM) to counteract hydrophobic mismatch (Bavi et al., 2017; Killian, 1998). Piezos have a total of 114 TMs, such that the sum of energetic contributions from hydrophobic constraints (estimated ∼1–2 kcal/mol per newly exposed residue; Chang et al., 2007; Moon and Fleming, 2011) might be particularly large. Testing whether membrane thinning is a major driver of gating will require the reconstitution of Piezos in lipid bilayers of systematically varying thicknesses and the measurement of gating energetics. Structural data may also reveal how membrane thickness affects Piezo conformations and gating transitions. Third, an increase in tension leads to an increase in area-per-lipid (Gullingsrud and Schulten, 2004). Indeed, lipids can be extruded from binding pockets upon increases in tension and subsequently result in pore opening—the “lipids-move-first” model for mechanosensitive gating (Brohawn et al., 2014b; Flegler et al., 2021; Zhang et al., 2021). While no lipids have been identified in pore pathways of currently available Piezo structures, a lipid-shaped density exists in the pocket between the anchor and the first helical bundle in one Piezo1 structure that may couple allosterically to pore closing (Saotome et al., 2018). Finally, as for many mechanosensitive ion channels, the intracellular membrane interface is lined with amphipathic helices that occur between most bundles (Box 1 and Fig. 1). In the “lipid dragging” model, lipids remain tightly associated with these helices during membrane expansion, resulting in lateral movements that could couple to pore opening (Bavi et al., 2017; Bavi et al., 2016a).

Clearly, force-from-lipids explains much of Piezo function and is sufficient as a sole source of energy for Piezo1 opening. It remains unknown, however, if and to what extent other mechanistic principles, including the force-from-tether model (see The force-from-tether model) and the membrane footprint model (see The membrane footprint model), also serve as significant sources of mechanical energy.

## The force-from-tether model

In the force-from-tether mechanism, mechanical energy is supplied by the application of a force (*F*) to a so-called gating spring, which is a structural domain with an elastic constant (*k*) that increases in length by an increment, Δ*x*. In a perfectly elastic system, the energy associated with this displacement is

$$∆G=-\frac{1}{2}∙k∙{∆x}^{2}\text{.}$$

Stretching and compression of the gating spring can be actuated by a tether that couples either directly to the membrane or to other cellular structures (i.e., the cytoskeleton or extracellular matrix). Importantly, the fact that a channel associates with a tether-like protein need not imply that gating is directly controlled by a spring; membrane-associated tethers can also affect local tension and thereby modulate channel gating via this mechanism.

Several mechanically activated ion channels have previously been shown to be directly gated via a tether mechanism. These include the *Drosophila* channel NompC, in which the gating spring is formed by intracellular ankyrin repeats that are compressed by microtubules with a spring constant of ∼13 pN/nm (Wang et al., 2021), as well as the mechanotransduction complex of hair cells, in which the channel pore (likely TMC1/2) is gated by a spring with a stiffness of 0.5 pN/nm (Howard and Hudspeth, 1988; Peng et al., 2011; Holt et al., 2021).

For Piezo1, several lines of evidence argue against a substantial contribution from a tether force toward gating energy. Most notably, the channel is mechanically activated in a cell-free system, indicating that an extrinsic tether is not required for channel gating (Syeda et al., 2016). Moreover, upward pulling on every accessible extracellular loop via magnetic particles with a force of ∼10 pN normal to the membrane failed to directly activate Piezo1 (Wu et al., 2016). Finally, Piezo1 channels diffuse freely and rapidly (∼0.05 μm^2^/s) across the membrane in several cell types, suggesting they are not tied to any cellular structures (Ellefsen et al., 2019; Ridone et al., 2020).

However, there is also contrasting evidence suggesting that Piezos may have the ability to couple to tether-like structures. In some cell types, Piezo1 may be tethered to the actin cytoskeletal network via interactions with E-cadherin, as disruption of this putative interaction reduces mechanosensitive currents (Wang et al., 2022). Additionally, extracellular matrix proteins sensitize Piezo1, particularly to pulling forces (Gaub and Muller, 2017). For Piezo2, the situation is even less clear. Its ability to respond to mechanical stimuli in a cell-free system has not yet been directly tested, and Piezo2 is less sensitive to pressure-clamp stimulation, which implies it may be less sensitive to membrane tension, i.e., force-from-lipids (see also Box 2). Moreover, an extracellular protein tether was previously identified to be critical for rapidly adapting mechanosensitive currents in dorsal root ganglion neurons—a current later identified to be carried by Piezo2 (Li and Ginty, 2014; Schwaller et al., 2021; Hu et al., 2010).

If a gating spring does directly contribute to Piezo channel gating energy, where might it be located? The central permeation pathway in both Piezo isoforms leads to three intracellular lateral portals (one per monomer) that have been proposed to be gated by a “plug and latch” mechanism in which the beam serves as a lever connected to the portals via latch and plug domains (Fig. 1; Geng et al., 2020; Taberner et al., 2019; Wang et al., 2019). In this framework, the plug, which is unresolved in the structure, obstructs the exit portals, and the latch is an attractive candidate for serving as the spring. Additionally, many of the extensive intracellular loops in both Piezo1 and Piezo2 have yet to be structurally resolved, which will help generate additional hypotheses about their ability to act directly as a spring or indirectly as a point of contact for intracellular tethers (Verkest et al., 2022). Interestingly, intracellular loops are also the site of alternative splicing for both isoforms, which may provide a cell-specific modulation of the contribution of intracellular tethers (Szczot et al., 2017; Geng et al., 2020).

In addition to directly transmitting force to the channel, intra- and/or extracellular tethers may indirectly alter Piezo gating by modulating local membrane tension and/or bending stiffness. Interestingly, given the variable rate of long-distance tension propagation among cells (Shi et al., 2018; Gomis Perez et al., 2022), a complementary tether-activated mechanism may allow fast and long-range transmission of otherwise local perturbations in membrane tension. Consistent with this idea, an intact cytoskeletal network is required for efficient activation of Piezo1 by traction forces, and it facilitates responses of Piezo1 to the poke assay (Gottlieb et al., 2012; Romero et al., 2019; Ellefsen et al., 2019). On the other hand, disruption of the cytoskeleton facilitates pressure-clamp–induced Piezo activation, suggesting that the cytoskeleton may also buffer mechanical energy and shield the channel from small mechanical perturbations (Retailleau et al., 2015; Cox et al., 2016). Thus, whether the cytoskeleton plays an amplifying or a mechanoprotective role may depend not only on cell type and local membrane composition, but also the nature of the stimulus.

## The membrane footprint model

In addition to the two canonical models of mechanosensitive ion channel gating discussed above, the unique size and shape of the Piezo structure has led to a third complementary model: gating via membrane footprint. The large, dome-shaped structure of Piezo1 is predicted to not only induce membrane curvature in regions of direct interaction with the lipid bilayer, but also deform the cell membrane well beyond the perimeter of the channel, resulting in a large membrane footprint (Haselwandter and Mackinnon, 2018). This bending of the cell membrane by Piezo will make an additional energetic contribution (Δ*G*_*M*_) that depends on the membrane bending stiffness (*K*_*b*_), the curvature of the mid-bilayer surface (*c*_1_ and *c*_2_), tension (γ), and the decrease in in-plane area (Δ*A*) upon membrane deformation:

$${\Delta G}_{M}=\frac{1}{2}K_{b}\int {\left(c_{1}+c_{2}\right)}^{2}dA+\gamma ∆A\text{.}$$

Further, in a continuum model of a lipid bilayer, the characteristic decay length (λ) of this footprint can be predicted by only three properties: the basic shape of the Piezo dome (modeled as a bowl with a radius of ∼10 nm), the membrane bending modulus (*K*_*b*_ ∼ 20 *k*_*B*_*T* for biological membranes), and tension (γ = 0.1 *k*_*B*_*T*/nm^2^ for a membrane at rest):

λ=Kbγ.

With these values, a footprint with a characteristic decay length of 14 nm is predicted for Piezo1 in the absence of applied tension. Strikingly, this results in a footprint that extends far greater than the boundary of the channel itself! As tension increases, the energy associated with maintaining the footprint increases; that is, under tension, it takes more work to bend the membrane into the shape of Piezo1’s footprint. Therefore, an increase in tension will favor a flatter conformation of the protein (with a correspondingly smaller footprint). Importantly, the tension-dependent contribution of the membrane footprint (Δ*G*_*M*_) to the total gating energy will sum with the energy required to form the dome against membrane tension $(∆G_{D}^{\gamma }=-\gamma ∙\Delta A\text{;}$ see The force-from-lipid model). Theoretical calculations predict that in typical biological membranes, these energies will contribute to similar degrees, although this will depend to some extent on local membrane properties; the footprint mechanism will provide a larger contribution in stiffer membranes, where greater work is required to bend the bilayer (Haselwandter and Mackinnon, 2018).

Is there experimental evidence that flattening of Piezo is coupled to channel opening? A study using high-speed atomic force microscopy to apply a compressive force to Piezo1 channels while simultaneously scanning their topography revealed they indeed flatten reversibly (Lin et al., 2019). Recent molecular dynamics simulations based on a partial Piezo1 structure are also consistent with the idea that flattening of the blades could couple to pore opening (De Vecchis et al., 2021; Jiang et al., 2021). This property may not be unique to Piezos, either: recent closed- and open-state structures of MSL1, a plant homolog of MscS, suggest that it undergoes a transformation from a bowl-like structure in the closed state that expands and flattens upon opening (Deng et al., 2020).

Importantly, the membrane footprint theory unifies some of the otherwise contradictory observations about Piezo gating, including the effects of the cytoskeleton and the relative contributions of force-from-lipids and force-from-tether. It allows the large size and curvature of Piezo to produce a large Δ*A*, and thus exquisite tension sensitivity, without a correspondingly large change in pore diameter, which would not be consistent with its small and selective pore. Additionally, Piezo will be sensitive to the size of local membrane compartments, which are formed by cytoskeletal attachments and will impose additional constraints on the size of its footprint (Krapf, 2018; Haselwandter and Mackinnon, 2018). Piezos will also be sensitive to the bending modulus of the membrane itself: in principle, a stiffer membrane bending modulus will yield lower tension thresholds for activation, though experimental data suggest that stiffer membranes may instead inhibit Piezo1 via an unknown mechanism (Romero et al., 2019; Zheng et al., 2019b). Finally, Piezo will also be highly sensitive to local curvature induced in the membrane by other proteins, which will create further energetic constraints on the size and shape of the Piezo footprint, as well as potentially bias the localization of Piezos toward domains with similar membrane curvature. Altogether, local variations in membrane composition and cytoskeletal organization will have a large contribution to Piezos’ precise sensitivity to an applied stimulus, which will vary not only with cell type, but also within different compartments of the same cell (discussed further in Heterogeneity of mechanical gating). This may help explain why one ion channel can perform such diverse roles (sensing shear stress, compression, flow, etc.) in many cell types.

## Piezo channel cooperativity

Nearby proteins can influence each other’s behavior via cooperativity, a phenomenon that has been well established for other ion channels, including voltage-gated calcium (Ca_V_) and hyperpolarization-activated cyclic nucleotide-gated (HCN) channels (Dekker and Yellen, 2006; Moreno et al., 2016). The extent of cooperativity depends on the local density of channels (the extent of clustering) as well as the degree of functional coupling. Do Piezo channels localize in close proximity in vivo? In keratinocytes, Piezo1 is spatially enriched at the retracting edge of wounds (Holt et al., 2021). In red blood cells, Piezo1 forms submicrometer clusters that preferentially localize near areas of higher tension (Dumitru et al., 2021). Punctate distributions of Piezo1 have also been observed in overexpression systems (Ridone et al., 2020), although the correlation between punctate fluorescence and number of channels per puncta has yet to be established. Is there any evidence for an effect of Piezo channel density on function? One recent study showed that the number of Piezo1 channels in a patch was positively correlated with an increase in resting open probability as well as a shift of the gating curve to lower pressures (Tharaka Wijerathne et al., 2021 *Preprint*). Work from our own laboratory showed the opposite result: channel density had no effect on tension sensitivity and vanishingly little influence on open probability in the nominal absence of membrane tension (Lewis and Grandl, 2021a). Clearly, more work needs to be done to establish the extent of functional cooperativity in additional cell types.

**Figure 5.**
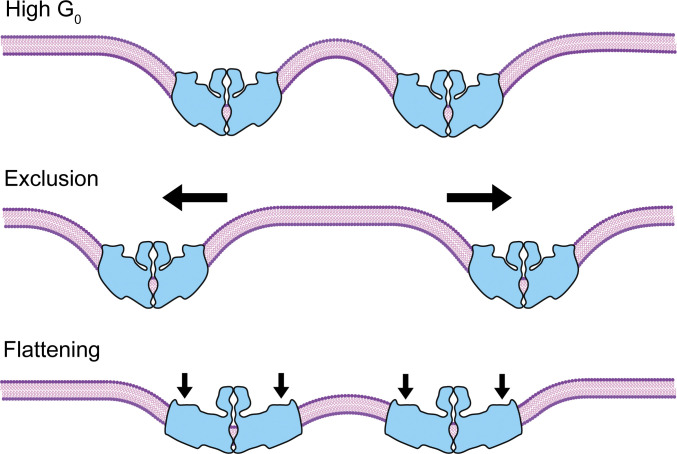
**Interaction between Piezo ion channels.** Top: Schematic showing two Piezo channels (blue) in proximity. When channels approach one another, the high degree of curvature resulting from their extended footprints will result in an energetic penalty. Middle: The overall energy of the system can be reduced by repulsion of nearby channels, resulting in a local exclusion zone. Bottom: Alternatively, the overall energy of the system can be minimized by flattening of the Piezo channel blades, reducing the high local curvature induced by their footprints.

What are the potential mechanisms for any effect of Piezo channel density on its function? One predicted consequence of Piezo’s large membrane footprint is that nearby channels would influence each other’s behavior: as two channels approach, the opposing curvature of their footprints will create a large energetic constraint on the intervening membrane (Fig. 5). This constraint could be dealt with in two ways: first, channels could spatially segregate to prevent footprint overlap; that is, channels could repel each other. Second, if opening is indeed coupled to flattening of the blades and a corresponding reduction of footprint size, then nearby channels might increase each other’s open probability, or in the extreme case, channels could undergo “tensionless gating” (Jiang et al., 2021).

In addition to footprint-mediated effects on cooperativity, the area expansion (Δ*A*) of any opening channel will decrease the overall membrane tension stress on all other channels. This buffering effect is inherent to all tension-gated membrane proteins and would result in a negative cooperativity that scales with channel density (Boucher et al., 2009). The extent to which Piezo channels buffer membrane tension depends on their area expansion and channel density: experimental evidence suggests this is small (Δ*A* = 6–20 nm^2^ per channel), although structural data suggest up to a doubling of area (∼500 nm^2^) is theoretically possible (Lewis and Grandl, 2015; Cox et al., 2016; Wang et al., 2019). Moreover, in biological membranes, other proteins may also undergo significant tension-induced area expansion, further sensitizing the function of Piezo channels to their overall environment.

## Modulation of Piezo mechanosensitivity via interaction of mechanical force with other physical stimuli

As discussed above, many ion channels are polymodal, responding cooperatively to multiple types of stimuli. In contrast, Piezos are particularly specialized to open in response to mechanical force—and not much else. However, in the presence of mechanical force, Piezos are subject to some degree of modulation by additional physical stimuli, which we will discuss briefly here.

Some Piezo isoforms (zebrafish and fly, as well as inactivation-deficient mouse Piezo1) are capable of transitioning to a voltage-dependent gating mode. Importantly, this mechanism requires a preceding mechanical stimulus, as well as outward permeation, and therefore substantially differs from canonical voltage-gating (Moroni et al., 2018). Once open, all Piezo channels are modulated by voltage: inactivation and deactivation kinetics are both slowed by depolarization (Coste et al., 2010; Lewis and Grandl, 2020). Interestingly, fast changes in voltage may also indirectly activate Piezo ion channels: action potentials have been shown to induce small but perceptible movements in the cell membrane (∼0.2–0.4 nm) that may lead to local perturbations in membrane tension (Yang et al., 2018).

Temperature modulates the gating of Piezo channels via multiple mechanisms. First, as for all channels, thermal energy (*G*_*thermal*_ = *k*_*B*_*T*) contributes directly to overcome Δ*G*. Temperature also indirectly modulates gating through its effects on membrane properties; specifically, warmer temperatures will thin the bilayer and decrease its bending modulus (Pan et al., 2008). Interestingly, temperature has a differential effect on Piezo1 versus Piezo2, perhaps hinting at differing contributions of lipid–membrane interactions to the gating mechanism (Box 2). Finally, as for all ion channels, temperature modulates the diffusion of ions in aqueous solution and thereby the unitary conductance, with a Q_10_ of 1.6 (Hille, 2001).

To date, the only ligands identified that modulate Piezos are Yoda1 and Jedi1/2, which are small molecules specific to Piezo1 (Syeda et al., 2015; Wang et al., 2018). Yoda1 lowers the tension threshold of the channel for opening, potentially by acting as a molecular wedge that promotes blade flattening (Syeda et al., 2015; Lacroix et al., 2018; Botello-Smith et al., 2019). No ligands have yet been identified to act on Piezo2, again speaking to the high specialization of these channels for sensing mechanical forces.

## Heterogeneity of mechanical gating

**Figure 6.**
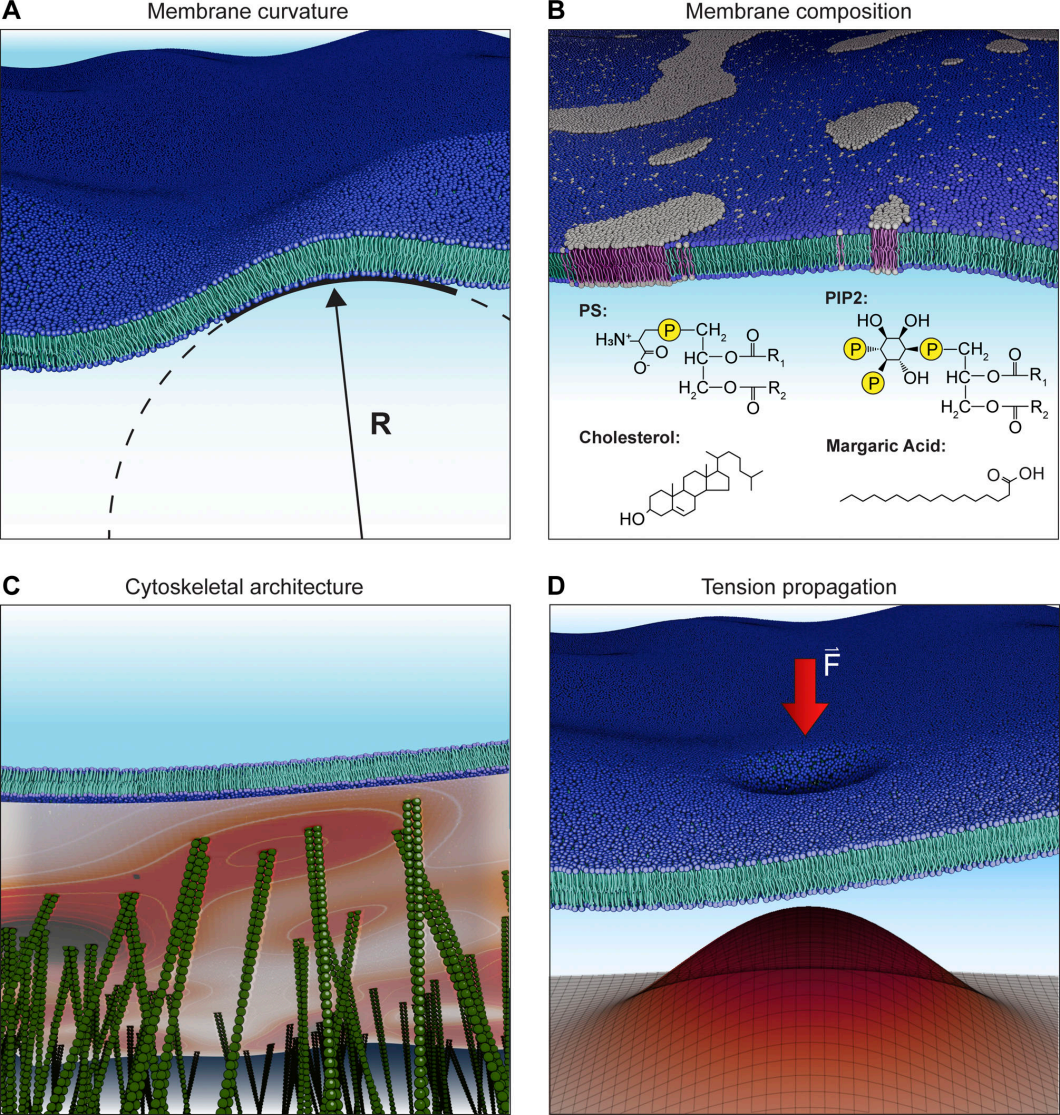
**Heterogeneity in mechanical environments. (A)** Membrane curvature. The radius of curvature (R) varies locally based on local density of Piezo ion channels and other membrane proteins. **(B)** Membrane composition. Lipids are unevenly distributed throughout a cell; in addition, heterogeneity in membrane leaflets and lipid macrodomains, such as lipid rafts, will result in cellular regions in which Piezos may be preferentially distributed and/or differentially sensitive to mechanical force. Structures of lipid species shown to modulate Piezo channel function are also shown (PS, phosphatidylserine; PIP2, phosphatidylinositol 4,5-bisphosphate). **(C)** Cytoskeletal architecture. The actin cortical network is tightly coupled to the plasma membrane, and the density of this coupling can vary spatially. The heatmap illustrates local cytoskeletal contact density in the schematized image, with higher densities shown in darker red. The density of this coupling will affect local mechanical properties and tension propagation. **(D)** Tension propagation. In many cells, tension propagation is slow, resulting in nonuniform stimulus intensity across the membrane when a force is locally applied. The magnitude of tension is schematized in the surface plot. Darker red and z-height represent higher tension.

Compared with other stimuli, which are relatively spatially homogeneous (e.g., temperature) or propagate relatively rapidly (e.g., voltage and ligands), mechanical forces are spatially and temporally heterogeneous: both cellular properties and the spatial distribution of channels will affect the overall mechanical response of a cell (Katta et al., 2019; Fig. 6). For example, lipid composition, which can differ substantially on short length scales, likely has large effects on Piezo gating through its effects on membrane properties, including stiffness. Indeed, margaric acid, cholesterol, phosphatidylserine, and phosphoinositides have all been shown to influence Piezo gating (Romero et al., 2019; Romero et al., 2020; Ridone et al., 2020; Borbiro et al., 2015; Tsuchiya et al., 2018). Similarly, the extent of cytoskeletal coupling to the channel and/or membrane may lead to heterogeneity in Piezo gating. This could occur indirectly, through the effects of the cytoskeleton on membrane compartment size, or directly, if a cytoskeletal protein binds the channel and acts as a tether. In addition, the synchrony of Piezo gating is affected by inactivation as well as the limited diffusion of membrane tension, the latter of which has been shown to be modulated by local membrane protein concentration (Shi et al., 2018)*.* Each of these effects will result in an overall broadening of the tuning curve: while the stimulus–response curve of an individual channel may be steep, local membrane dynamics will shift the midpoint of activation for each individual channel, as well as the time course of activation, resulting in an overall shallower response (Figs. 4 and 6).

## Looking forward: Future directions in Piezo gating energetics

One major gap in knowledge of Piezos is that no structures have captured a pore that is sufficiently dilated to permit ion flow, and therefore all are assumed to be in a closed or inactivated state. An open-state structure is critical for illuminating the precise conformational changes that occur during the gating cycle, which in turn will provide valuable insight into mechanism. For example, comparison of closed and open states will reveal whether flattening results in a dilated pore, as well as the true extent of area expansion. Several avenues could help achieve this goal. As has classically been done for many channels, gain-of-function mutations or addition of agonists may stabilize the open state (Lewis and Grandl, 2020; Zheng et al., 2019a; Syeda et al., 2015). Alternatively, the protein could be reconstituted in nanodiscs altered to have artificially high tension or low curvature, thus promoting opening. Two avenues for doing so include incorporation of cyclodextrins (Cox et al., 2021) or light-actuated lipids (Doroudgar et al., 2021).

Another major gap is a precise quantification of Piezo activation kinetics: while the equilibrium occupancy of closed and open states depends only on their respective free energies, the rate at which equilibrium is reached (i.e., the speed of opening) depends on the activation energy. Quantification of activation kinetics would give key insight into what types of stimuli the channels might be best suited to transduce; for example, if Piezos are tuned to sense vibrations of specific frequencies, including those relevant for hearing (Lewis et al., 2017; Li et al., 2021). Existing methods for activating the channels are slower than the presumed time constant of activation. Both piezo-actuated cellular indentation (“poke”) and pressure clamp (“stretch”) require ∼10 ms to reach maximum amplitude (Lewis and Grandl, 2021b); consequently, both methods are too slow to resolve the speed of Piezo gating. New technologies, such as the use of photonic force or ultrasound, continue to emerge and will be crucial for precise measurements of gating kinetics (Abeytunge et al., 2021; Sorum et al., 2021; Prieto et al., 2018).

Standard force application techniques are also insufficient to fully probe Piezos, as they are unspecific and/or lack quantification. Specifically, the stretch assay, while allowing relatively precise application of pressure, only allows for control of global curvature (Bavi et al., 2016b). Moreover, it fails to robustly activate Piezo2 (Box 2). Thoroughly testing the membrane footprint hypothesis for both isoforms will require the ability to locally clamp curvature and/or tension. The poke assay robustly activates both Piezo1 and Piezo2; however, it produces a nonhomogeneous force of unknown amplitude that likely rapidly dissipates away from the time and point of indentation, such that the ensemble of channels will fail to reach *P*_*o*_ ≈ 1. In a recently developed assay, cells are plated on elastomeric micropillars that are deflected to locally stimulate channels at the cell-substrate interface, but this method still only allows for control of pillar deflection amplitude, and not the magnitude of force acting on the cell or channel (Poole et al., 2014). A full characterization of Piezo gating kinetics will be facilitated by the ability to precisely quantify force and simultaneously measure activity, for example, by combining electrophysiology with atomic force microscopy (Gaub and Muller, 2017); even better would be locally clamping force (Eastwood et al., 2015). Finally, to directly probe the tether mechanism, optical traps or magnetic tweezers could be used to mechanically probe domains identified to serve as putative springs (Basu et al., 2016).

A more complete understanding of the energetics and activation mechanisms for Piezo would be useful for later exploitation of the process: for example, it would facilitate engineering of a remotely activatable ion channel via magnetic force (Wu et al., 2016; Lee et al., 2021). More importantly, it would enable us to understand how cells detect and integrate mechanical energy to act as force sensors.
